# Pleural plaques: only an imagiologic finding?

**DOI:** 10.11604/pamj.2019.34.156.20227

**Published:** 2019-11-21

**Authors:** Paulo Almeida, Ana Araújo

**Affiliations:** 1 Internal Medicine Service, Baixo Vouga Hospital Center, Aveiro, Portugal

**Keywords:** Asbestos, pleural diseases, occupational exposure

## Image in medicine

An 80-year-old man who worked as a construction worker (specifically involved in the placement of roofs and ceilings) between the ages of 25 and 60 years was admitted due decompensated heart failure. The chest radiograph (CRX) showed cardiomegaly, signs of bibasal congestion and well delimited focal areas suggestive of pleural calcifications (A). A high-resolution chest computerized tomography (HRCT) was revealed multiple gross calcifications, some of them associated with pleural thickening areas suggestive of pleural plaques (PP) (B, C). The pulmonary function tests, bronchofibroscopy and pleural biopsies had no alterations. We initiated diuretics and optimized disease-modifying heart failure therapy with improvement and resolution of the symptoms. Asbestos exposure is a well-known risk factor for thoracic malignancies and non-malignant respiratory diseases, such as asbestosis, PP and diffuse pleural thickening. PP are one of the earliest and the commonest manifestation of asbestos exposure affecting up to more than half individuals in occupational setting. PP are considered to be a marker of exposure and, typically, they are seen 20-30 years after asbestos dust inhalation. PP are usually asymptomatic, but they can cause decline in lung function. Such as the presented patient, the diagnosis relies on radiographic findings and a compatible history of exposure. A CRX is used as the standard diagnostic method, but this procedure has important limitations in the detection of early subtle PP, whereas a HRCT enables diagnosis of thin or tiny noncalcified plaques. PP do not require specific treatment. Even being asymptomatic, our patient kept follow-up in ambulatory.

**Figure 1:**
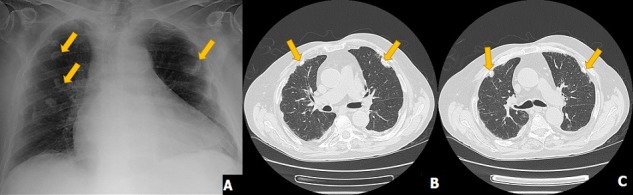
(A) chest radiograph (CRX) revealed showed cardiomegaly, signs of bibasal congestion and well delimited focal areas suggestive of pleural calcifications; (B, C) high-resolution chest computerized tomography (HRCT) revealed multiple gross calcifications in axial sections with windows to the mediastinum, some of them associated with pleural thickening areas suggestive of pleural plaques (PP), without pulmonary parenchyma changes

